# Microenvironmental Regulation of Tumor Progression and Therapeutic Response in Brain Metastasis

**DOI:** 10.3389/fimmu.2019.01713

**Published:** 2019-07-24

**Authors:** Michael Schulz, Anna Salamero-Boix, Katja Niesel, Tijna Alekseeva, Lisa Sevenich

**Affiliations:** ^1^Institute for Tumor Biology and Experimental Therapy, Georg-Speyer-Haus, Frankfurt, Germany; ^2^Biological Sciences, Faculty 15, Goethe University, Frankfurt, Germany; ^3^Frankfurt Cancer Institute, Goethe University, Frankfurt, Germany; ^4^German Cancer Consortium (DKTK, Partner Site Frankfurt/Mainz) and German Cancer Research Center (DKFZ), Heidelberg, Germany

**Keywords:** brain metastases, tumor microenviroment, microglia, astrocytes, immune system, immunotherapy, neurons

## Abstract

Cellular and non-cellular components of the tumor microenvironment (TME) are emerging as key regulators of primary tumor progression, organ-specific metastasis, and therapeutic response. In the era of TME-targeted- and immunotherapies, cancer-associated inflammation has gained increasing attention. In this regard, the brain represents a unique and highly specialized organ. It has long been regarded as an immunological sanctuary site where the presence of the blood brain barrier (BBB) and blood cerebrospinal fluid barrier (BCB) restricts the entry of immune cells from the periphery. Consequently, tumor cells that metastasize to the brain were thought to be shielded from systemic immune surveillance and destruction. However, the detailed characterization of the immune landscape within border-associated areas of the central nervous system (CNS), such as the meninges and the choroid plexus, as well as the discovery of lymphatics and channels that connect the CNS with the periphery, have recently challenged the dogma of the immune privileged status of the brain. Moreover, the presence of brain metastases (BrM) disrupts the integrity of the BBB and BCB. Indeed, BrM induce the recruitment of different immune cells from the myeloid and lymphoid lineage to the CNS. Blood-borne immune cells together with brain-resident cell-types, such as astrocytes, microglia, and neurons, form a highly complex and dynamic TME that affects tumor cell survival and modulates the mode of immune responses that are elicited by brain metastatic tumor cells. In this review, we will summarize recent findings on heterotypic interactions within the brain metastatic TME and highlight specific functions of brain-resident and recruited cells at different rate-limiting steps of the metastatic cascade. Based on the insight from recent studies, we will discuss new opportunities and challenges for TME-targeted and immunotherapies for BrM.

## Introduction

The stepwise process in which cancer cells disseminate from the primary tumor site to colonize distant organs is biologically a highly inefficient process, yet metastasis accounts for 90% of cancer related deaths (1). In particular, metastasis to the brain represents a considerable burden and is associated with high morbidity and unfavorable prognosis for patients (2). A central question in the biology of metastasis remains the preference of certain tumor types to colonize individual organs, such as the brain. Gene signatures that mediate the preferential organ tropism have been identified (3). Differentially expressed genes in tumor cell variants with high tropism for a specific organ are often associated with factors that assist tumor cells to overcome tissue specific barriers, e.g., the blood brain barrier (BBB), or to generate a cancer permissive niche in potentially hostile environments (4, 5). In addition to tumor cell intrinsic traits, the ability of tumor cells to rapidly co-opt niche cells in foreign organs to exploit their functions and to block or evade anti-tumor activity is a key determinant for successful metastatic colonization (6, 7).

Upon entry into the central nervous system (CNS), tumor cells are confronted with the highly complex and specialized brain tissue environment that is fundamentally different from the primary site with respect to cellular constituents, matrix composition, metabolism, and immune landscape (6). The cellular composition of the brain is represented by the main functional cells, including neurons and auxiliary cell types, macroglia (astrocytes and oligodendrocytes), and microglia. In addition to brain resident cell types, blood-borne immune and inflammatory cells have recently gained attention as potent mediators of brain metastasis-associated inflammation. While the presence of tumor-infiltrating lymphocytes is often correlated with better prognosis and is indicative for higher response rates to immunotherapy, high content of myeloid cells is associated with immune suppression, tumor promotion, and therapy resistance (8). In this review, we highlight the complex interactions between tumor cells and tumor-associated niche cells and discuss current knowledge on cell type-specific pro- or anti-tumor functions of cells in the tumor microenvironment (TME) in brain metastases (BrM). Based on this knowledge, we will discuss opportunities and challenges for TME-targeted or immunotherapies against BrM.

### Neurons in Brain Metastases—Innocent Victims or Critical Mediators?

**Figure 1:**
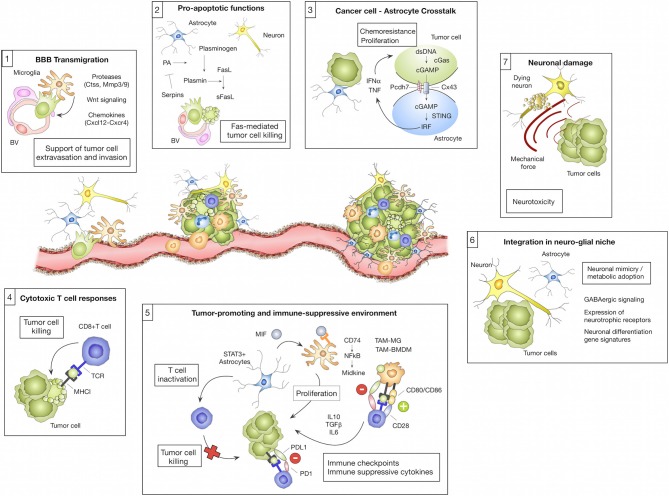
Microenvironmental regulation of the metastatic cascade. The tumor microenvironment of brain metastasis comprises different brain-resident and recruited cell types with cell-type and/or stage-dependent pro- or anti-tumor functions. (1) Different microglial-derived factors including proteases (e.g., Ctss, Mmp3, and Mmp9), Wnt signaling components or chemokines (e.g., Cxcl12) have been implicated in assisting tumor cells to cross the blood brain barrier (BBB) and colonize the brain parenchyma. (2) In contrast, astrocytes were shown to prevent early stages of metastatic colonization by inducing soluble (s)-FasL-mediated tumor cell killing. Tumor cell-derived serpins can block this effect by inhibiting astrocyte-derived plasminogen activator (PA), therefore preventing the generation of active plasmin that converts FasL into sFasL. (3) While the initial tumor cell—astrocyte contact leads to tumor cell killing, close interactions between tumor cells and astrocytes via gap junctions foster tumor cell proliferation and protect tumor cells from chemotherapy. This process was linked to the transfer of cGAMP from tumor cells to astrocytes that triggers cGas-STING-mediated IRF activation leading to production of IFNα and TNF. (4) Cytotoxic T cells represent an important component of the adoptive immune response against brain metastasis by executing tumor cell killing. (5) However, T cell activity is efficiently blunted by the immune-suppressive milieu in brain metastasis. T cell activity is modulated through interaction with several cell types including tumor cells, tumor-associated macrophages/microglia (TAM-MG and TAM-BMDM) and astrocytes by expressing immune checkpoint molecules or by secreting immune-suppressive cytokines (e.g., IL10, TGFβ or IL6). Moreover, astrocytes with high STAT3 expression were shown to activate tumor-promoting TAMs via the MIF-CD74-NfkB-Midkine axis. (6) Tumor cells that colonize the brain were shown to adopt to the neuro-glial niche by acquiring neuronal gene signatures that induce specific metabolic programs (e.g., GABAergic signaling and the expression of neurotrophic factors). (7) Tumor expansion leads to neuronal damage by mechanical compression of neurons.

Neurons, as highly specialized cells responsible for cell-to-cell signal propagation, are certainly one of the most critical and highly abundant cell types in the CNS (9). However, to date little is known about their contribution to BrM. Currently, astrocytes and microglia, as well as recruited peripheral immune cells, are within the main focus of research in the context of BrM. Neurons are mostly regarded as passive bystanders and neuronal cell death and dysfunction are rather thought to result from collateral damage in the process of BrM progression and/or treatment. Neuronal cell death results from persistent neuro-inflammation caused by reactive microglia and astrocytes in response to tumor cells. Myelinating glial cells and oligodendrocytes are also functionally compromised in this tumor-reactive milieu and thus further contribute to neuronal dysfunction (10). Interestingly, glial dysfunction and its effect on myelin sheath development are implicated in common side effects of chemotherapy. Those characteristic cognitive symptoms are collectively referred to as chemobrain (11). Moreover, a recent study by Seano et al. shed additional light on the cause of neuronal cell death in the presence of BrM. The authors demonstrated that mechanical compressive stress from a solid tumor leads to indirect neuronal malfunction and blood vessel degeneration in the peri-tumor area thereby causing neuronal cell death by critical deformation of the neuronal bodies (Figure 1; Boxes 6, 7). Intriguingly, the authors were able to show that common neuroprotective lithium medication was effective in preventing neuronal damage and alleviate in part negative cognitive symptoms (12).

While the niche cells in the CNS have to cope with the arrival and expansion of tumor cells, also metastatic cancer cells have to adapt to the brain microenvironment, which differs considerably from the tissue of origin (6). The extent of this adaptation has been demonstrated by Neman et al. (13). The authors show that breast cancer cells are capable to change their metabolic machinery and to mimic the reciprocal relationship between neurons and astrocytes by expressing all major genes of a GABAergic phenotype, a feature attributed to neurons (13) (Figure 1; Box 6). This adaptive mechanism allows cancer cells to utilize a novel energy source, glutamate, prevalent in the normal brain. A follow up study by Schnepp et al. has shown that this feature is not exclusive to breast cancer cells. The authors unveiled the mechanism of this genetic shift, implicating increased GABA synthesis by metastatic cancer cells via methylation-dependent upregulation of glutamate decarboxylase 1 (GAD1) expression (14). Interestingly, Schnepp et al. have shown that this precise feature can be used to explore novel treatment options, such as GABA antagonists, frequently used for seizure treatment. While it is increasingly recognized that tumor cells have to adopt to the unique metabolism of the brain in order to thrive, it is less well-characterized to which extent metastatic tumor cells that arise from epithelial origin can benefit from neuronal growth factors as previously demonstrated for primary brain cancers. Glioma, as primary brain cancer, arise from different neuronal or glial cell lineages (i.e., neural stem/progenitor cells or oligodendroglial lineage) (15) and hence originate from cells that are known to be influenced by neuronal activity (16, 17). Indeed, it has been shown that neuronal excitation and subsequent release of synaptic adhesion protein Neuroligin-3 (NLGN3), Brain Derived Neurotrophic Factor (BDNF), and neurotransmitter such as dopamine and serotonin are utilized by glioma cells to promote tumor growth (18–20). Moreover, it has been shown that glioma cells can influence neuronal excitation in the vicinity of tumors through secretion of glutamate, thus ensuring the supply of proliferative factors (18, 21). Interestingly, although breast-to-brain metastatic tumor cells are of epithelial origin, there is evidence that breast cancer cells express receptors for two major neurotrophic growth factors, neuronal growth factor (NGF) and BDNF (22). Moreover, a recent transcriptome analysis of tumor- and stromal signatures in BrM revealed an enrichment of neuronal differentiation pathways in the tumor cell population (23). Further exploration of neuronal mimicry revealed that GABAergic signaling is not limited to the CNS, but has also emerged as a tumor signaling molecule in cancers of peripheral organs such as breast, liver, pancreas, and colon (24). Hence, it is possible that tumor cells are primed for GABAergic signaling already at the primary tumor site providing an advantage for rapid adaptation to metabolic conditions in the brain. Moreover, it was demonstrated that prostate cancer cells induce axonogensis and use growing axons as migratory tracts for cancer cell dissemination (19, 25).

To date it remains unknown to which extent neurons play an active role in BrM onset and progression. However, given the recently demonstrated role of neurons in glioma together with the observation that highly innervated tumors (i.e., prostate or head and neck cancer) are more aggressive than their less innervated counterparts (18, 19, 25), it might be premature to exclude neurons as active players in BrM. Future studies will hopefully provide more detailed insight into the role of neurons in BrM and potentially open new therapeutic avenues against BrM by targeting interactions between tumor cells and neurons.

### Astrocytes in Brain Metastases—Versatile Players in Mediating Distinct Steps Within the Brain Metastatic Cascade

Astrocytes belong to the glial cell types and represent the most abundant cell population within the CNS (26). Originally described as star-like “glue” cells of the CNS, the variety and complexity of astrocyte function in health and disease is increasingly recognized. Under normal conditions their role in tissue homeostasis includes maintenance of the blood brain-barrier (BBB), immune signaling, regulation of extracellular ion, and fluid homeostasis, as well as control and maintenance of a broad range of functions implicated in modulating neuronal networks, such as regulation of synaptogenesis, synaptic plasticity, and elimination, neurotransmitter clearance, and neurotrophin secretion (27–30). To fulfill this functional diversity, it is now widely accepted that astrocytes represent a highly heterogeneous cell population (28, 31–33). With the advent of high-throughput single cell sequencing and other “*omic*” approaches, the existence of several astrocyte subpopulations was revealed in rodents (31, 34, 35). An even higher heterogeneity was found within the human brain (36, 37). Interestingly, neuronal stimuli have been shown to determine distinct features of astrocytes (38). Moreover, it was shown that during aging, astrocytes change their transcriptomes in different regions of the murine brain (39), which is in part orchestrated by interacting with local microglia (40). Given the phenotypic and functional diversity of astrocytes, it is not surprising that astrocytes play a central role in maintaining tissue homeostasis and in regulating neuro-glial communication under physiological conditions. Consequently, astrocytes are also often found to be involved in disease progression of different CNS malignancies (30). Moreover, malignantly transformed astrocytes are the cell of origin for astrocytoma, the most common form of glioma (41). Astrocytes respond to disease-associated stimuli by undergoing morphological and functional changes, which are collectively referred to as reactive astrogliosis (30, 42, 43). A key feature of reactive astrocytes (RA) is the formation of a glial scar that confines pathological foci from the healthy parenchyma (27). Interactions between astrocytes and other brain-resident or recruited cells have been investigated in different disease models. It was shown that every cell type in the CNS can release factors that induce astrogliosis (27), and the outcome is regulated in a time- and context-dependent manner (44).

A particularly close connection between astrocytes and microglia was observed in the diseased CNS (10). Depending on the environmental stimuli, astrocytes acquire different activation states that are referred to as A1 and A2 following the previously defined nomenclature to classify macrophage polarization states into pro-inflammatory, anti-tumor M1 macrophages, and immune-suppressive, tumor-promoting M2 macrophages (45, 46). A1 astrocytes are regarded as neuro-inflammatory, while A2 astrocytes are associated with neuro-protective features by promoting survival and growth of neurons and by inducing repair mechanisms (43). However, it should be noted that the M1/M2 and A1/A2 nomenclature reflects functional and phenotypic extremes within a spectrum of activation states and that along the continuum of activation states mixed phenotypes have been reported (47). The high phenotypic plasticity of astrocytes was also reported in pre-clinical and clinical studies on different neurodegenerative disorders. For example, Liddelow et al. demonstrated that microglia induce A1 astrocytes with neurotoxic properties. The presence of A1 astrocytes was demonstrated in various human malignancies from the spectrum of neurodegenerative diseases (10). On the contrary, microglia were shown to exert crucial functions in activating neuroprotective astrocytes in a model of *Spinal Cord Injury* (SCI) (48), further underpinning the context-dependent outcome of cellular interactions. In line with this finding, microglia-mediated blockade of an A1 astrocyte conversion was shown to be neuro-protective in a mouse model of sporadic Parkinson's Disease (49). There is also evidence that RA are regulated by distinct T cell subsets in neuro-inflammatory conditions such as stroke, which then potentiates neurological recovery (50).

While our understanding of astrocyte function in neurodegenerative disorders is steadily increasing, we are just at the beginning to decipher the underlying mechanisms of pro- or anti-tumor functions of astrocytes in BrM (51, 52). Induction of astrogliosis is an early event during metastatic colonization and outgrowth. This early reaction is attributed to neuro-protection by delineating metastatic foci from the normal brain parenchyma. Valiente et al. proposed that early contacts between tumor cells and astrocytes lead to tumor cell death and clearance of the majority of tumor cells that enter the brain. In order to successfully colonize the brain, tumor cells have to acquire traits to block pro-apoptotic stimuli from astrocytes (53) (Figure 1; Box 2). On the other hand, there is accumulating evidence that astrocytes promote distinct steps of the metastatic cascade, including initial seeding and support of tumor outgrowth (54–56). Moreover, astrocytes have been shown to protect tumor cells from chemotherapy (57). This process was shown to be dependent on gap junction formation (57, 58). The importance of direct cellular connections between astrocytes and breast- or lung brain metastatic tumor cells via gap junctions was further demonstrated by Chen et al. (59). In this context, gap junction formation was mediated by connexin43 (Cx43) and protocadherin (Pcdh7) and activated the innate immune response pathway cGAS-Sting (Cyclic GMP-AMP synthase-stimulator of interferon genes) leading to secretion of tumor-supportive cytokines such as IFNα and TNF (Figure 1; Box 3). Functional co-option of RA by melanoma cells was further exemplified by Schwartz et al. (60). The authors demonstrated in a melanoma brain metastasis model that astrogliosis is exploited by the tumor cells to support their growth (60). Astrocytes are also emerging as critical modulators of immune responses in BrM by interacting with brain-resident and recruited inflammatory cells. Priego et al. recently proposed an important role of astrocytes in the modulation of innate and acquired immunity in BrM (61). The authors identified a subpopulation of RA with high STAT3 activation levels associated with BrM of different primary origin. STAT3 activation was shown to affect microglia and T cell functions, likely leading to the establishment of an immunosuppressive microenvironment (Figure 1; Box 5). CD74+ TAMs were previously shown to generate an immunosuppressive milieu by reducing the secretion of IFNγ in glioma (62). More recently it was demonstrated in BrM that CD74+ TAMs depend on pSTAT3+ astrocytes that secrete macrophage migration inhibitory factor (MIF), the ligand for CD74. In response to ligand binding, CD74 acts as a transcription factor and promotes the expression of NFkB downstream targets, such as midkine, a factor that promotes cell viability (61). MIF inhibition by ibudilast led to a reduction of BrM in organotypic cultures (61). Moreover, genetic and pharmacological inhibition of STAT3 resulted in impaired viability of tumor cells and reduced outgrowth of brain metastasis (61). Heiland et al. recently confirmed the findings on STAT3+ astrocytes in primary brain tumors and demonstrated that astrocyte-microglia interactions generate a strong immune-suppressive environment due to up-regulation of PD-L1 on tumor-associated astrocytes and production of cytokines such as IL10 and TGFβ (63).

Taken together, astrocytes are emerging as one of the key regulators of brain metastatic colonization and outgrowth. Owing to their high phenotypic and functional heterogeneity, astrocytes exert pro-tumor as well as anti-tumor functions. Detailed insights into the existence of different astrocyte subpopulations or stimuli that polarize astrocytes at distinct stages of the brain metastatic cascade are required to develop astrocyte-targeted therapies.

### Myeloid Cells in Brain Metastases—Origin and Location Matters

Myeloid cells in brain malignancies comprise a highly abundant and heterogeneous cell population and consist of brain resident myeloid cells as well as recruited cells including monocytes, bone marrow-derived macrophages (BMDM), and granulocytes (64). Brain-resident microglia are the major representatives of the innate immune system in the CNS and exert critical functions in immune surveillance and host defense. In addition to functions related to neuro-inflammation, microglia are also responsible for synapse pruning and remodeling (65). Microglia represent a unique cell type among the glial cells with respect to their ontological origin. In contrast to other glial cells, microglia are of mesodermal origin and arise from primitive hematopoietic progenitors (erythromyeloid progenitors) that are present in the yolk sac during embryonic development (66–68). In addition to parenchymal microglia, the CNS harbors myeloid cell populations that reside in specific regions of the CNS including the choroid plexus, the interphase between blood and meninges, and the perivascular space of vessels (69–71). Border-associated macrophages (BAMs) derive from erythro-myeloid precursors that arise from the yolk sac and the fetal liver. Interestingly, bone marrow-derived monocytes also contribute to the choroid plexus macrophage population (70–72). Moreover, monocytes have been shown to reside within the meninges (73). BAMs are believed to have a higher antigen presenting capacity than microglia, largely due to higher expression of MHCII (74), however their contribution to BrM progression remains to be elucidated. Detailed insight into transcriptional programs of microglia revealed a remarkable plasticity in response to a wide variety of stimuli, such as regional differences in the brain, aging, sex, or the composition of the microbiome and gene signatures reflect cellular functions during developmental stages (75–78). In addition to in depth analysis of microglial heterogeneity, dissecting gene signatures of disease-associated microglia provides detailed insight into lineage-dependent functions and cellular dynamics (79–82). In this regard, the identification of a disease-associated signature in microglia (DAM) by single cell sequencing in Alzheimer's disease, aging, multiple sclerosis, and amyotrophic lateral sclerosis models significantly contributed to our understanding how different pathological conditions shape the molecular identity of disease-associated cells and how the respective subpopulation might enhance or ameliorate disease progression. Upregulation of phagocytosis components and neurodegenerative markers such as Trem2 and ApoE and the downregulation of microglia homeostatic markers such as Cx3cr1 and Tmem119 were shown to be characteristic for the DAM signature (71, 79–82). Remarkably, single cell analysis of human microglia from multiple sclerosis patients revealed an even higher heterogeneity, as seven different populations of microglia were identified. Three of those populations represented homeostatic genes, one population showed an upregulation of chemokine and cytokine signaling, whereas the three other populations correlated with the clusters associated with demyelination and remyelination in mice (83). Although the BrM field currently lacks detailed insight into the molecular identity of disease-associated macrophages/microglia compared to neurodegenerative diseases or primary brain tumors, a series of pre-clinical studies shed light into tumor-associated macrophage (TAM) functions during distinct steps of the metastatic cascade. Invasion of metastasizing tumor cells is rapidly sensed by microglia and the presence of single tumor cells is sufficient to recruit and to activate microglia (84, 85). Given the role of microglia in immune surveillance and host defense, it is tempting to speculate that the initial contact between tumor cells and microglia at sites of extravasation leads to clearance of invading tumor cells. However, Chuang et al. demonstrated that tumor cells block pro-apoptotic functions of microglia and exploit tissue damage responses to increase their invasive capacity (86). The role of microglia in tumor cell extravasation was further confirmed by Qiao et al. using a CSF1R inhibitor to deplete microglia in prevention trial settings in a mouse model for melanoma BrM (84). The authors also found that Mmp3 expression by microglia was negatively correlated with ZO-1 expression on endothelial cells. Moreover, the incidence of melanoma BrM was decreased by Mmp3 inhibition (84). Co-option of microglial functions and adoption of leukocytic characteristics to increase the capacity of tumor cells to colonize the brain parenchyma was previously proposed (5) (Figure 1; Box 1). Interestingly, it was observed that tumor cells increase the expression of cathepsin S, a protease that is pre-dominantly expressed by leukocytes, to cleave junctional adhesion molecules that maintain the BBB integrity and thus assist tumor cells to breach the BBB. Importantly, only the combined depletion of cathepsin S in the tumor and stroma compartment was efficient to reduce BrM burden (5). The co-option of leukocyte characteristics by tumor cells is also evident in the role of the C-X-C chemokine receptor type-4 (Cxcr4) along with its ligand Cxcl12 that are involved in lymphocyte chemotaxis. Cxcr4 expression has been detected in BrM tumor cells (87, 88). Remarkably, the inhibition of this pathway decreased breast cancer cell migration (89) and impaired BrM establishment (90).

In established BrM, tumor-associated macrophages and microglia are the most abundant non-cancerous cell type and constitute up to 30% of the total tumor mass (5). In primary brain cancer, TAMs tend to be pro-tumorigenic and accumulate with higher tumor grade (91, 92). As revealed by immunohistochemistry of BrM sections, microglia and macrophages showed signs of intratumoral activation and formed a boundary between the tumor mass and normal brain tissue (93–95). They were identified as foamy cytoplasmatic cells with shortened cell processes and immunoreactive to CD68. However, there is currently no clinical evidence in BrM for a correlation between microglia density and activation marker expression with treatment modality, anatomic brain regions or necrosis (96). Despite the lack of clinical correlation between TAM content and BrM patient prognosis, pre-clinical data indicate tumor-promoting functions of TAMs in BrM. The crosstalk between microglia and melanoma BrM is evident from the alteration of JNK and p38 components in microglia, which may attenuate their phagocytic response, as well as ERK and STAT3 in melanoma cells, which are linked to angiogenesis. The authors also provided evidence for a metastasis-supportive niche, as secretion of vascularization factors was reshaped and proliferation of both cell types was increased (97). Correlating with the latter finding, anti-inflammatory microglia depletion by mannosylated clodronate liposomes decreased the growth of intracranially implanted breast cancer cells (98). Another evidence from the interplay between cancer cells and microglia is that XIST-deficient-breast cancer cells led to an increased amount of M2-markers in microglia (99). However, the genetic programs that lead to an induction of tumor-promoting functions in TAMs in BrM are not well-characterized to date. Detailed analysis of signaling pathways and transcription factor activity is required to evaluate if similar mechanisms lead to the induction of a TAM gene signature in BrM as proposed for other tumor types including glioma (64, 100). For example, Blazquez et al. recently proposed the importance of PI3K signaling as a master regulator of tumor promoting activation states of macrophages/microglia (101).

Previous studies that interrogated the role of TAMs in BrM did not discriminate between cells originating from brain-resident microglia or from bone marrow-derived macrophages. As mentioned earlier, under steady-state conditions, bone marrow-derived myeloid precursors do not contribute to the microglia pool. However, damage to the blood-brain barrier as described for BrM (102–104) allows the recruitment of such progenitors that supplement the microglial population (105). In this context it is important to evaluate to which extent the integrity of the BBB has to be diminished in order for blood-borne cells to efficiently breach the BBB. It was shown that the BBB in BrM is not fully disrupted but rather remodeled into a blood-tumor-barrier (BTB) due to alterations in the pericyte subpopulation (106). While this is not sufficient to allow free penetration of therapeutic antibodies or chemical compounds that are not BBB permeable (107), it is possible that vessel structures of the BTB lose their capability of restricting the entry of blood-borne immune cells and at the same provide the necessary molecular structures such as adhesion molecules for efficient transmigration of peripheral leukocytes. Cell-tracing techniques based on the transplantation of genetically labeled HSCs into mice following whole-body irradiation or head protected irradiation have been used to decipher the origins of TAMs in primary brain tumors (108). By means of transplantation and lineage-tracing models, numbers for peripheral macrophages range between 25 and 75% in glioma and 25% in BrM (64, 92, 108). Similar to neurodegenerative disorders, the bulk FACS sorted TAMs showed a different expression profile compared to normal microglia and monocytes in a mouse glioma model (64). More importantly, the profile of tumor-associated microglia and macrophages was different, confirming the functional impact of their different ontological origin. While TAM-MG showed profiles rich in cytokines, chemokines, and complement components, TAM-BMDM signatures were associated with wound healing, antigen presentation and immune suppression (64, 92). Another evidence for the intrinsic differences within the macrophage/microglia population is the lack of impact of anti-CSF1 treatment on microglia compared to monocyte-derived cells, which may be due to the presence of the CSF1R alternate ligand IL34 (109). This observation is also supported by the fact that in multiple sclerosis dendritic cells (DC) and monocyte-derived cells are the major antigen presenting cells (APCs). Indeed interactions of microglia with infiltrating T cells were found to be transient (110). Importantly, in order to unravel the molecular pathways and functional pre-dominance in every cell population, it is mandatory to properly distinguish them. Under physiological conditions, the different expression profiles of macrophages and microglia, residing in their respective environment enables their differentiation (69, 110–112). The identification of novel markers for microglia such as Tmem119, P2ry12, Sall1, SiglecH (113–116) is important to unravel their specific role in health and disease. However, these expression patterns are less well-defined in TAMs, as e.g., homeostatic Tmem119 is upregulated in TAM-BMDM, while it is downregulated in TAM-MG (64). Remarkably, CD49d has been described as a differential marker between blood-borne macrophages and microglia in brain malignancy (64). However, to date gene expression signatures of TAM-MG in comparison to TAM-BMDM have not been investigated.

In summary, within the myeloid compartment in BrM, TAMs constitute the most abundant cell population. Based on their ontological origin and localization within the brain parenchyma and border regions of the CNS, they represent a highly heterogeneous cell population. Until now, most pre-clinical studies that aimed to unravel the role of TAMs in BrM did not discriminate between different subpopulations. The identification of lineage-restricted markers that allow to distinguish TAM-MG and TAM-BMDM as well as single cell sequencing approaches will help to unravel gene signatures of individual subpopulations and provide insight into their functional contribution in BrM.

### Tumor-Infiltrating Lymphocytes in Brain Metastases—Can Activity be Unleashed Without Inducing Neurotoxicity?

Traditionally, the brain has been regarded as an immune privileged organ, with lack of peripheral immune surveillance through blood-borne immune cells such as T cells owing to the blood-brain-barrier (BBB) and the lack of effective lymphatic drainage (117, 118). However, this view has recently changed, as it is recognized that while the brain might be privileged to some extent, this does not mean total exclusion of blood-borne immune cells. Clearly, the entry to the parenchyma is strictly controlled to prevent fatal neurotoxicity, but patrolling leukocytes, such as bone marrow-derived antigen-presenting DC as well as CD4+ and CD8+ T cells, have been identified in the meninges and choroid plexus (70, 71, 77, 119, 120). DC have a higher capacity of antigen presentation and T cell stimulation than microglia (121). Furthermore, it has been demonstrated that afferent antigen sampling from the brain parenchyma does take place and CNS-derived antigens can lead to peripheral priming of T cells (122). Moreover, recent studies unveiled the existence of lymphatic vessels in the meninges, which can transport antigens, derived from the brain parenchyma via the cerebrospinal fluid/lymphatic system into the deep cervical lymph nodes (123–125), indicating that the brain has a functional draining lymphatic system. However, the details about anatomy and composition, as well as the efferent route of T cells into the brain, specifically in the scenario of BrM, require further investigation. As immunotherapies gain increasing attention, the infiltration of BrM with T cells and their function in the context of brain tumors comes into focus. By now, several studies demonstrated that tumor infiltrating T lymphocytes (TILs) are present in BrM of different primary cancers such as Non-Small Cell Lung Cancer (NSCLC), Small Cell Lung Cancer (SCLC), renal cell cancer (RCC), melanoma, or breast cancer. Of these RCC and melanoma show the highest CD3+ numbers and highest CD8+/CD3+ ratio. The infiltration patterns of TILs seem to be diverse, ranging from a diffuse spreading through the metastases to an accumulation in the stroma and around vessels, depending on primary tumor type (126). The prognostic value of TIL numbers is currently being disputed, with several studies indicating favorable outcome on survival (96, 127, 128), while Harter et al. could not find a significant correlation (126). Moreover, Mustafa et al. demonstrated that T cells promote breast cancer BrM. This is due to a direct interaction of T cells with tumor cells, leading to increased guanylate binding protein 1 (GBP)-1 expression by the latter, which in turn enables them to cross the BBB (129). These conflicting data indicate that further research is necessary to elucidate the complex function of T cells in BrM as well as possible influence of the TME on T cells. It is conceivable, that the latter might be polarized in the TME under certain conditions resulting in tumor promoting rather than anti-tumor functions, so they will not only be inhibited in their anti-tumor functions but promote tumor growth as discussed for macrophages and astrocytes in the sections above. Furthermore, it remains unclear what dictates the number of TILs in BrM. Generally, T cells are primed in peripheral lymph nodes (e.g., cervical lymph nodes). Extravasation and T cell homing to sites of inflammation or tissue injury is dependent on binding of VLA-1 and LFA-1 expressed by T cells to endothelial cellular adhesion molecules (CAMs) such as VCAM-1 or ICAM-1 (130–132). The exact entry route for T cells into BrM is not yet fully understood, however there is evidence that expression of CAMs on endothelial cells plays a central role in the homing process of T cells. For example, it was shown that vessels in the proximity of tumor lesions show expression of VCAM-1, ICAM-1, and other CAMs in different BrM models (133–136). Interestingly, it was proposed that tumor cells exploit this mechanism to breach the BBB and home to the brain parenchyma (134). Serres et al. could also detect VCAM-1 on human BrM samples, while healthy controls showed only minimal expression (133). Additionally, it was demonstrated that VCAM-1 expression increases with tumor progression in a BrM mouse model (133). Taggert et al. proposed that increased CD8+ T cell trafficking to BrM is dependent on VCAM-1 and ICAM-1 expression induced by IFNγ produced by BMDMs, microglia and NK cells (135). Hence, TIL numbers can at least in part be determined by IFNγ levels and CAM expression. However, other determinates of TIL numbers in BrM such as mutational load and presence of tumor antigens are expected to affect T cell infiltration. It was demonstrated for primary melanoma, a highly immunogenic tumor with high TIL content, that the density of antigens did not correlate with the presence or absence of TILs (137). Additionally, Mansfield et al. applied TCR profiling of patient derived samples and could show that the mutational burden is higher in BrM of NSCLC than in the respective primary tumor and is correlated with T cell richness (138). In this study the authors also observed a contraction of T cell clones compared to the primary site, with the 10 most abundant T cell clones being more heavily expanded compared to the primary tumor site (138). This expansion hints toward an immune response in BrM with the involvement of antigen specific T cells, even though in a more restricted manner than in primary tumors. The fact that BrM still represents a highly aggressive, fatal disease and monotherapies with immune modulatory agents only show modest effects indicates that this adaptive immune response is not strong enough to halt tumor growth. Comparison with the respective primary tumors led to the observation that BrM, e.g., derived from breast cancer, have a lower content of TILs than the primary counterpart, with 5 and 20%, respectively (139). While the extent of T cell exclusion is lower in BrM compared to many primary brain tumors, the TME is still highly immune suppressive. Current research investigates strategies to increase the inflammatory response against BrM, to render the tumors more prone to immune-modulating agents. Using adoptive T cell transfer is only one strategy, which has demonstrated encouraging results in melanoma BrM patients (140). However, not only the number of T cells plays an important role in the immune response against brain metastatic cells, but also their activation status is relevant. The latter is dependent on many factors and the cell composition in the TME. The brain naturally constitutes an immune suppressive microenvironment to prevent fatal neurotoxicity, potentially resulting in the exhaustion and inactivation of T cells in primary brain tumors (141). Moreover, it has been demonstrated with different BrM mouse models, that the number of FOXP3^+^ T regulatory cells (Tregs) is increased during BrM progression. These results have also been recapitulated on patient samples from melanoma and NSCLC BrM (142). Additionally, not only the tumors themselves are infiltrated with Tregs, but also the blood of patients bearing BrM contains an increased percentage of Tregs compared to healthy donors (143). Those inhibitory T cells can hypothetically contribute to the exhaustion of anti-tumor effector T cells.

Taken together, insights into the complex and dynamic interplay between different cell types of the TME in BrM, in which the activity of individual cell populations is tightly controlled by other cell types, underpins the challenges in developing effective therapies against BrM. However, a comprehensive view on the complex interactions provides opportunities for the development of improved therapeutic intervention strategies as discussed in the following paragraph.

### Tumor Microenvironment-Targeted and Immunotherapies Against Brain Metastases

**Figure 2:**
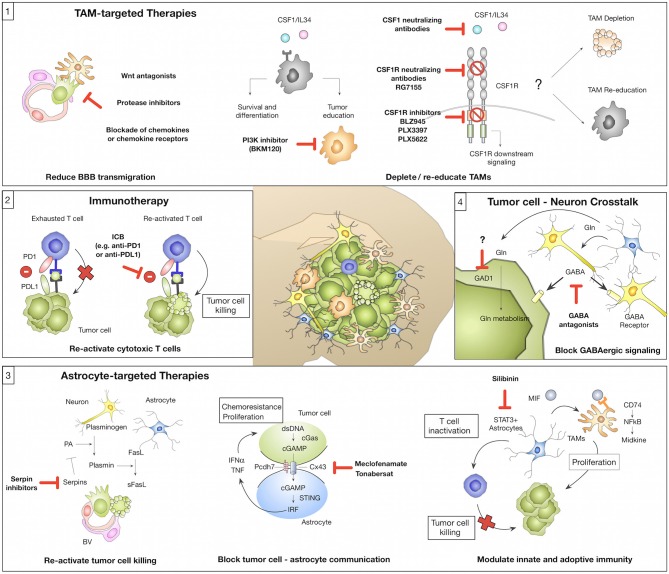
Novel concepts of tumor microenvironment-targeted therapies or immunotherapies (1) Tumor-associated macrophages/microglia (TAMs) represent a highly abundant cell type in BrM with known roles in mediating tumor cell BBB transmigration and tumor-supportive functions that foster metastatic outgrowth. Strategies for TAM-targeted therapies include the reduction of tumor cell BBB transmigration (e.g., by Wnt antagonists, protease inhibitors, or blockade of chemokines/chemokine receptors). Blockade of CSF1-CSF1R signaling represents another strategy to target TAMs by inhibiting a central pathway for macrophage differentiation and survival. The CSF1-CSF1R signaling axis can be inhibited by (i) CSF1 blocking antibodies (with no effects on IL34 mediated CSF1R activation), (ii) CSF1R blocking antibodies, or (iii) ATP competitive small molecule inhibitors. Consequences of CSF1R inhibition on TAMs in established BrM (depletion vs. re-education) remain to be elucidated. An alternative strategy might be the inhibition of Pi3K by BKM130 to prevent the activation of pro-tumor TAMs. (2) Tumor-infiltrating T cells in BrM show signs of T cell exhaustion mediated by immune checkpoints (e.g., PD1-PDL1) or immune-suppressive cytokine milieus. Blockade of immune checkpoints e.g., by anti-PD1 or anti-PDL1 reactivates T cells and reinstates tumor cell killing by cytotoxic T cells. (3) Astrocytes represent a highly plastic cell type in BrM and their function was associated with pro- and anti-tumor activity. Inhibition of serpins could re-activate sFasL-mediated tumor cell killing and thereby prevent early metastatic colonization. Blockade of gap junctions by meclofenamate or tonabersat was shown to inhibit tumor cell-astrocyte crosstalk that supports proliferation and protects tumor cells from chemotherapy. Targeting of STAT3+ astrocytes by silibinin represents a strategy to block the induction of pro-proliferative functions of TAMs and reduce astrocyte-mediated inactivation of T cells. (4) Brain metastatic tumor cells adopt neuronal features to integrate into the neuro-glial niche and to exploit brain specific energy sources e.g., glutamate (Gln). GABA antagonists were shown to reduce GABAergic signaling in tumor cells. Furthermore, blockade of Gln influx into tumor cells by GAD1 inhibition could represent a promising therapeutic strategy.

The development of effective therapies against BrM is one of the most challenging aspects of cancer research. Intervention strategies developed for extracranial tumors cannot easily be translated into effective therapeutic avenues for brain cancers. Instead, approaches have to be tailored to the unique brain environment to breach tissue-specific restrictions of therapeutic efficacy, but at the same time consider the protection of delicate anatomical structures that control higher cognitive functions. Detailed insights into the critical cellular and molecular drivers of BrM are necessary to provide a scientific rationale for the development of improved intervention strategies. Recent research efforts shed light on the complexity of the tumor-stroma crosstalk in BrM and indicate potential therapeutic targets for immune- or tumor microenvironment targeted therapies (Figure 2).

#### Immunotherapies Against Brain Metastases

The introduction of immunotherapy has recently revolutionized treatment options for a range of extracranial primary tumor types including melanoma and NSCLC that frequently metastasize to the brain. Hence, it appears logical to test the efficacy of immunotherapy against BrM, even though the brain tissue environment represents one of the most immune suppressed milieus. One arm of immunotherapy aims at re-activating T effector cells via immune checkpoint inhibition (Figure 2; Box 2). Indeed monoclonal antibodies, which block immune checkpoints (e.g., anti-CTLA4, anti-PD1, or anti-PDL1), demonstrate efficacy in individual BrM patients, but the overall response rates are modest, even in melanoma BrM, which is thought to be highly immunogenic (144–147). For example, a limited number of retrospective and prospective clinical trials indicate intracranial response rates of 16–25% following ipilimumab treatment in melanoma patients (148, 149) and 50–55% in trials combining ipilimumab and nivolumab (ABC trial and CheckMate 204) (150, 151). Therefore, other strategies are being explored to increase T cell immunity e.g., the combination of checkpoint inhibition with radiotherapy (RT). The latter has the potential to sensitize for immune modulation by inducing immunogenic cell death resulting in secretion of inflammatory cytokines, upregulation of MHCI and therefore increased trafficking of T cells to the BrM, as shown for other cancers (152, 153). Taggart et al. could show in a mouse model of melanoma BrM that successful immunotherapy depends on enhanced trafficking of CD8+ T cells, activated in peripheral lymphoid organs, to the brain parenchyma (135). Additionally, RT can potentially increase the tumor mutational load thereby broadening the immune response (154). Indeed, radio-immunotherapies show promising results and are currently being tested in clinical trials also for BrM (155). Nevertheless, T effector cell activity in the brain is not only dependent on Treg infiltration or immune suppressive cytokines in the TME, but also on the presence of APCs. As mentioned earlier, in the brain this role can be fulfilled by DC, BMDM, and to a lesser extent by microglia (100, 121). The presence of those cell types in the brain tumor context is not questioned anymore, but there are no detailed reports about specific interaction with APCs and TILs that lead to antigen-specific T cell activation in BrM. It is important to further investigate this in the future to improve response rates of patients to immunotherapy and to find new strategies against BrM by exploiting the full potential of T cell immunity in this context. Using DC vaccines to boost T cell responses is only one of many potential treatment possibilities, which could be explored in this context and is under current investigation in brain tumors (156). Another strategy of applying T cells for BrM treatment is the delivery of genetically engineered CAR T cells directed against known tumor antigens, which led to reduced tumor growth in a xenograft mouse model (157). Currently, this approach is investigated in a clinical trial for breast cancer patients with BrM (158). However, it remains questionable if T cell-directed therapies can be successful in the presence of a highly immune suppressive myeloid compartment. Alternatively, one could argue that myeloid-targeted therapies might be more promising.

#### Modulating the Myeloid Compartment in Brain Metastases

Cells of the myeloid compartment represent the most abundant non-malignant cell type in the BrM microenvironment. Pre-clinical data indicate a critical role in mediating distinct steps within the metastatic cascade leading to the generation of a cancer-permissive, immune suppressive environment (Figure 1). Different strategies have been employed to target TAMs in BrM to evaluate therapeutic efficacy. Blocking macrophage survival and differentiation by disrupting CSF1-CSF1R signaling represents one of the most promising strategies (Figure 2; Box 1). Since there are two cognate ligands that bind to CSF1R, targeting the receptor rather than the ligand, leads to efficient blockade of CSF1R downstream signaling. CSF1R inhibition can be achieved by CSF1R blocking antibodies (e.g., RG7155) (159) or ATP competitive small molecule inhibitors (e.g., BLZ945, PLX3397, or PLX5622) (91, 160) (Figure 2; Box 1). Qiao et al. employed PLX3397 in a prevention trial setting and demonstrated that microglia depletion reduced tumor cell transmigration potential of melanoma brain metastatic cells (84). This is in line with previous findings that demonstrated that clodronate liposome mediated microglia depletion resulted in a reduction of the BrM burden (98). Given the promising results of TAM-targeted therapies with the CSF1R inhibitor BLZ945 in a mouse model of pro-neural glioblastoma (91) it remains to be elucidated whether CSF1R inhibition in established BrM shows anti-tumor activity. Importantly, analyses in two independent glioblastoma models revealed that conditions in which CSF1R inhibition leads to TAM depolarization show higher efficacy compared to TAM depletion (91, 161). Consequently, research effort should be put on the identification of gene signatures that determine tumor-promoting vs. anti-tumor characteristics in TAMs to specifically target tumor supportive traits of TAMs but spare physiologically important functions. Blazquez et al. recently proposed Pi3K signaling as a master regulator of tumor-promoting functions of BrM-associated macrophages/microglia and demonstrated that BKM120, a pan-PI3K inhibitor, reduced tumor-promoting features of macrophages/microglia (101). However, it is important to note that clinical data revealed better overall survival for patients with high PI3K activity, while patients with moderate or low PI3K activity showed worse prognosis (101). Hence, inhibiting PI3K signaling in BrM might have opposing effects depending on which cell type is targeted.

Given the importance of the myeloid compartment to establish an immune suppressive environment to protect the CNS from neuro-inflammation, myeloid-targeted therapies should be taken into account carefully. Blocking an integral part of a tissue protective mechanism might unleash unwanted pro-inflammatory responses that lead to detrimental tissue damage. Detailed knowledge in disease-associated effector functions of different myeloid cell populations is therefore needed to block tumor-promoting functions but maintain critical functions in host defense and neuro-protection.

#### Astrocyte-Targeted Therapies

Astrocytes are emerging as one of the key regulators of BrM (51). However, pre-clinical studies revealed high functional heterogeneity with tumor-promoting and anti-tumor functions. Therefore, it will be critical to gain detailed mechanistic insight into functional subpopulations or conditions that favor the induction of anti- vs. pro-tumor functions. Pre-clinical studies provided critical insight into potential therapeutic targets for astrocyte-targeted therapies. Valiente at al. demonstrated that tumor cells successfully block Fas- mediated cell killing by blocking the activity of plasminogen activator via serpins (53). Neutralizing tumor-derived serpins could therefore reinstate tumor cell killing during early metastatic colonization (Figure 2; Box 3). However, from a clinical perspective, strategies that control established disease are more urgently needed. One possibility is the blockade of astrocyte-tumor cell crosstalk via gap junctions to block tumor promotion. Chen et al. demonstrated that shRNA-mediated knockdown of Cx43 or Pcdh7 reduced the tumor burden and pharmacological intervention with the gap junction inhibitors meclofenamate and tonabersat decreased growth kinetics of BrM in pre-clinical trials (59) (Figure 2; Box 1). Although targeting of gap junctions shows promising results in pre-clinical disease models, the applicability of this approach in the clinic has to be carefully evaluated. Given the physiological importance of gap junctions for tissue integrity as well as normal brain function (162), potential adverse effects have to be taken into account. Moreover, approaches that target the formation of gap junctions between astrocytes and tumor cells are expected to be most efficient at initial stages of brain colonization, when the majority of tumor cells is in direct contact with astrocytes, while at later stages only tumor cells at the tumor-stroma interface are in close vicinity to astrocytes (30, 38, 51). Indeed, the formation of gap junctions between tumor cells and astrocytes was detected in subpopulations but not ubiquitously (59).

Another promising approach was recently described by targeting STAT3 signaling in RAs via the inhibitor Silibinin (61) (Figure 2; Box 3). Clinical data from lung cancer BrM patients treated with Silibinin showed significantly increased overall survival in response to STAT3 inhibition (61). However, some patients did not respond and the progression of extra-cranial disease was not affected, providing the possibility for BrM relapse. It remains to be shown how patients with BrM derived from other primary tumor entities respond to this treatment approach, and if variability of the outcome is due to tumor heterogeneity, differences in the TME and/or different patient histories. It is also unclear why only a subset of astrocytes activates STAT3 signaling, which requires deeper understanding, especially with respect to other immune cells (e.g., macrophages, microglia) and how different cellular and also molecular (e.g., different cytokine milieus) microenvironments influence the outcome of impaired STAT3 signaling.

Targeting astrocytes in the context of BrM is a promising approach, since these cells are highly susceptible to tumor cell-mediated education within the brain, thus promoting BrM. However, it remains to be investigated how distinct astrocyte subpopulations support BrM formation to develop strategies that block tumor-promoting or enhance anti-tumor functions of astrocytes.

#### Prevention of Neuronal Mimicry of Tumor Cells

Tumor cells that successfully colonize the brain fulfill certain criteria that allow them to integrate into the neuronal niche to evade immune destruction and to exploit brain specific energy sources to propagate their growth (Figure 1; Box 6). Strategies that prevent tumor cells from functionally integrating into the neural niche and to exclude them from important energy sources are expected to have critical clinical impact. For example, blockade of GABAergic signaling with GABA antagonists was proposed as a promising strategy to block the availability of glutamate as an energy source (13, 14) (Figure 2; Box 4). However, strategies that target traits that tumor cells acquire to hijack the tissue environment bear the risk of adverse effects by targeting physiologically highly relevant pathways. Future studies are therefore needed to understand mechanisms used by tumor cells to adopt to the neuronal-glial niche to interfere with the acquisition of neuronal-like features, rather than blocking cell-cell communication or metabolic pathways within the CNS.

## Concluding Remarks

The importance of the TME in BrM is increasingly recognized. In particular tumor immunology in BrM is an emerging field. While the brain was traditionally regarded as an immunological sanctuary site, it is now evident that BrM induce the recruitment of immune and inflammatory cells from the periphery and that routes for CNS-derived antigen presentation to peripheral immune cells exist. The presence of different brain-resident and recruited cell types in BrM opens new opportunities for TME-targeted interventions or immunotherapies. Recent studies that sought to unravel the functional contribution of different BrM-associated stromal cell types provide first insight into the complexity of tumor-stroma interactions as well as heterotypic signaling between niche cells that mutually modulate effector functions. Given the important role of the brain in controlling higher cognitive functions, it is particularly critical to consider a balance between the induction of anti-tumor responses and the maintenance of tissue protective mechanisms that prevent neurotoxicity. While we are just at the beginning to understand the complex interplay between different cells of the TME, more detailed insight is necessary to develop effective treatment strategies and to evaluate consequences of therapies that modulate effector functions within the BrM microenvironment.

## Author Contributions

MS, AS-B, KN, TA, and LS conceptualized and wrote the manuscript.

### Conflict of Interest Statement

The authors declare that the research was conducted in the absence of any commercial or financial relationships that could be construed as a potential conflict of interest.

## References

[1] Lambert AW Pattabiraman DR Weinberg RA. Emerging biological principles of metastasis. Cell. (2017) 168:670–91. 10.1016/j.cell.2016.11.037 28187288 PMC5308465

[2] Stelzer KJ. Epidemiology and prognosis of brain metastases. Surg Neurol Int. (2013) 4:S192–202. 10.4103/2152-7806.111296 23717790 PMC3656565

[3] Nguyen DX Massague J. Genetic determinants of cancer metastasis. Nat Rev Genet. (2007) 8:341–52. 10.1038/nrg2101 17440531

[4] Bos PD Zhang XH Nadal C Shu W Gomis RR Nguyen DX. Genes that mediate breast cancer metastasis to the brain. Nature. (2009) 459:1005–9. 10.1038/nature08021 19421193 PMC2698953

[5] Sevenich L Bowman RL Mason SD Quail DF Rapaport F Elie BT. Analysis of tumour- and stroma-supplied proteolytic networks reveals a brain-metastasis-promoting role for cathepsin S. Nat Cell Biol. (2014) 16:876–88. 10.1038/ncb3011 25086747 PMC4249762

[6] Sleeman JP. The metastatic niche and stromal progression. Cancer Metastasis Rev. (2012) 31:429–40. 10.1007/s10555-012-9373-9 22699312 PMC3470821

[7] Quail DF Joyce JA. Microenvironmental regulation of tumor progression and metastasis. Nat Med. (2013) 19:1423–37. 10.1038/nm.3394 24202395 PMC3954707

[8] Galon J Bruni D. Approaches to treat immune hot, altered and cold tumours with combination immunotherapies. Nat Rev Drug Discov. (2019) 18:197–218. 10.1038/s41573-018-0007-y 30610226

[9] von Bartheld CS Bahney J Herculano-Houzel S. The search for true numbers of neurons and glial cells in the human brain: a review of 150 years of cell counting. J Comp Neurol. (2016) 524:3865–95. 10.1002/cne.24040 27187682 PMC5063692

[10] Liddelow SA Guttenplan KA Clarke LE Bennett FC Bohlen CJ Schirmer L. Neurotoxic reactive astrocytes are induced by activated microglia. Nature. (2017) 541:481–7. 10.1038/nature21029 28099414 PMC5404890

[11] Gibson EM Nagaraja S Ocampo A Tam LT Wood LS Pallegar PN. Methotrexate chemotherapy induces persistent tri-glial dysregulation that underlies chemotherapy-related cognitive impairment. Cell. (2019) 176:43–55.e13. 10.1016/j.cell.2018.10.049 30528430 PMC6329664

[12] Seano G Nia HT Emblem KE Datta M Ren J Krishnan S. Solid stress in brain tumours causes neuronal loss and neurological dysfunction and can be reversed by lithium. Nat Biomed Eng. (2019) 3:230–45. 10.1038/s41551-018-0334-7 30948807 PMC6452896

[13] Neman J Termini J Wilczynski S Vaidehi N Choy C Kowolik CM. Human breast cancer metastases to the brain display GABAergic properties in the neural niche. Proc Natl Acad Sci USA. (2014) 111:984–9. 10.1073/pnas.1322098111 24395782 PMC3903266

[14] Schnepp PM Lee DD Guldner IH O'Tighearnaigh TK Howe EN Palakurthi B. GAD1 upregulation programs aggressive features of cancer cell metabolism in the brain metastatic microenvironment. Cancer Res. (2017) 77:2844–56. 10.1158/0008-5472.CAN-16-2289 28400476 PMC5461057

[15] Alcantara Llaguno S Sun D Pedraza AM Vera E Wang Z Burns DK. Cell-of-origin susceptibility to glioblastoma formation declines with neural lineage restriction. Nat Neurosci. (2019) 22:545–55. 10.1038/s41593-018-0333-8 30778149 PMC6594191

[16] Gibson EM Purger D Mount CW Goldstein AK Lin GL Wood LS. Neuronal activity promotes oligodendrogenesis and adaptive myelination in the mammalian brain. Science. (2014) 344:1252304. 10.1126/science.1252304 24727982 PMC4096908

[17] Mitew S Gobius I Fenlon LR McDougall SJ Hawkes D Xing YL. Pharmacogenetic stimulation of neuronal activity increases myelination in an axon-specific manner. Nat Commun. (2018) 9:306. 10.1038/s41467-017-02719-2 29358753 PMC5778130

[18] Venkatesh HS Johung TB Caretti V Noll A Tang Y Nagaraja S. Neuronal activity promotes glioma growth through neuroligin-3 secretion. Cell. (2015) 161:803–16. 10.1016/j.cell.2015.04.012 25913192 PMC4447122

[19] Venkatesh H Monje M. Neuronal activity in ontogeny and oncology. Trends Cancer. (2017) 3:89–112. 10.1016/j.trecan.2016.12.008 28718448 PMC5518622

[20] Gillespie S Monje M. An active role for neurons in glioma progression: making sense of Scherer's structures. Neuro Oncol. (2018) 20:1292–9. 10.1093/neuonc/noy083 29788372 PMC6120364

[21] Venkatesh HS Tam LT Woo PJ Lennon J Nagaraja S Gillespie SM. Targeting neuronal activity-regulated neuroligin-3 dependency in high-grade glioma. Nature. (2017) 549:533–7. 10.1038/nature24014 28959975 PMC5891832

[22] Termini J Neman J Jandial R. Role of the neural niche in brain metastatic cancer. Cancer Res. (2014) 74:4011–5. 10.1158/0008-5472.CAN-14-1226 25035392 PMC4122250

[23] Wingrove E Liu ZZ Patel KD Arnal-Estape A Cai WL Melnick MA. Transcriptomic hallmarks of tumor plasticity and stromal interactions in brain metastasis. Cell Rep. (2019) 27:1277–92.e7. 10.1016/j.celrep.2019.03.085 31018140 PMC6592283

[24] Young SZ Bordey A. GABA's control of stem and cancer cell proliferation in adult neural and peripheral niches. Physiology. (2009) 24:171–85. 10.1152/physiol.00002.2009 19509127 PMC2931807

[25] Magnon C Hall SJ Lin J Xue X Gerber L Freedland SJ. Autonomic nerve development contributes to prostate cancer progression. Science. (2013) 341:1236361. 10.1126/science.1236361 23846904

[26] Herculano-Houzel S. The human brain in numbers: a linearly scaled-up primate brain. Front Hum Neurosci. (2009) 3:31. 10.3389/neuro.09.031.2009 19915731 PMC2776484

[27] Sofroniew MV. Molecular dissection of reactive astrogliosis and glial scar formation. Trends Neurosci. (2009) 32:638–47. 10.1016/j.tins.2009.08.002 19782411 PMC2787735

[28] Miller SJ. Astrocyte heterogeneity in the adult central nervous system. Front Cell Neurosci. (2018) 12:401. 10.3389/fncel.2018.00401 30524236 PMC6262303

[29] Santello M Toni N Volterra A. Astrocyte function from information processing to cognition and cognitive impairment. Nat Neurosci. (2019) 22:154–66. 10.1038/s41593-018-0325-8 30664773

[30] Sofroniew MV Vinters HV. Astrocytes: biology and pathology. Acta Neuropathol. (2010) 119:7–35. 10.1007/s00401-009-0619-8 20012068 PMC2799634

[31] John Lin CC Yu K Hatcher A Huang TW Lee HK Carlson J. Identification of diverse astrocyte populations and their malignant analogs. Nat Neurosci. (2017) 20:396–405. 10.1038/nn.4493 28166219 PMC5824716

[32] Zhang Y Barres BA. Astrocyte heterogeneity: an underappreciated topic in neurobiology. Curr Opin Neurobiol. (2010) 20:588–94. 10.1016/j.conb.2010.06.005 20655735

[33] Cuevas-Diaz Duran R Wei H Wu JQ. Single-cell RNA-sequencing of the brain. Clin Transl Med. (2017) 6:20. 10.1186/s40169-017-0150-9 28597408 PMC5465230

[34] Zeisel A Hochgerner H Lonnerberg P Johnsson A Memic F van der Zwan J. Molecular architecture of the mouse nervous system. Cell. (2018) 174:999–1014.e22. 10.1016/j.cell.2018.06.021 30096314 PMC6086934

[35] Saunders A Macosko EZ Wysoker A Goldman M Krienen FM de Rivera H. Molecular diversity and specializations among the cells of the adult mouse brain. Cell. (2018) 174:1015–30.e16. 10.1016/j.cell.2018.07.028 30096299 PMC6447408

[36] Kelley KW Nakao-Inoue H Molofsky AV Oldham MC. Variation among intact tissue samples reveals the core transcriptional features of human CNS cell classes. Nat Neurosci. (2018) 21:1171–84. 10.1038/s41593-018-0216-z 30154505 PMC6192711

[37] Oberheim NA Takano T Han X He W Lin JH Wang F. Uniquely hominid features of adult human astrocytes. J Neurosci. (2009) 29:3276–87. 10.1523/JNEUROSCI.4707-08.2009 19279265 PMC2819812

[38] Farmer WT Abrahamsson T Chierzi S Lui C Zaelzer C Jones EV. Neurons diversify astrocytes in the adult brain through sonic hedgehog signaling. Science. (2016) 351:849–54. 10.1126/science.aab3103 26912893

[39] Boisvert MM Erikson GA Shokhirev MN Allen NJ. The aging astrocyte transcriptome from multiple regions of the mouse brain. Cell Rep. (2018) 22:269–85. 10.1016/j.celrep.2017.12.039 29298427 PMC5783200

[40] Clarke LE Liddelow SA Chakraborty C Munch AE Heiman M Barres BA. Normal aging induces A1-like astrocyte reactivity. Proc Natl Acad Sci USA. (2018) 115:E1896–905. 10.1073/pnas.1800165115 29437957 PMC5828643

[41] Jiang Y Uhrbom L. On the origin of glioma. Ups J Med Sci. (2012) 117:113–21. 10.3109/03009734.2012.658976 22348397 PMC3339543

[42] Khakh BS Sofroniew MV. Diversity of astrocyte functions and phenotypes in neural circuits. Nat Neurosci. (2015) 18:942–52. 10.1038/nn.4043 26108722 PMC5258184

[43] Liddelow SA Barres BA. Reactive astrocytes: production, function, and therapeutic potential. Immunity. (2017) 46:957–67. 10.1016/j.immuni.2017.06.006 28636962

[44] Colombo E Farina C. Astrocytes: key regulators of neuroinflammation. Trends Immunol. (2016) 37:608–20. 10.1016/j.it.2016.06.006 27443914

[45] Mantovani A Sozzani S Locati M Allavena P Sica A. Macrophage polarization: tumor-associated macrophages as a paradigm for polarized M2 mononuclear phagocytes. Trends Immunol. (2002) 23:549–55. 10.1016/S1471-4906(02)02302-5 12401408

[46] Murray PJ Allen JE Biswas SK Fisher EA Gilroy DW Goerdt S. Macrophage activation and polarization: nomenclature and experimental guidelines. Immunity. (2014) 41:14–20. 10.1016/j.immuni.2014.06.008 25035950 PMC4123412

[47] Martinez FO Gordon S. The M1 and M2 paradigm of macrophage activation: time for reassessment. F1000Prime Rep. (2014) 6:13. 10.12703/P6-13 24669294 PMC3944738

[48] Bellver-Landete V Bretheau F Mailhot B Vallieres N Lessard M Janelle ME. Microglia are an essential component of the neuroprotective scar that forms after spinal cord injury. Nat Commun. (2019) 10:518. 10.1038/s41467-019-08446-0 30705270 PMC6355913

[49] Yun SP Kam TI Panicker N Kim S Oh Y Park JS. Block of A1 astrocyte conversion by microglia is neuroprotective in models of Parkinson's disease. Nat Med. (2018) 24:931–8. 10.1038/s41591-018-0051-5 29892066 PMC6039259

[50] Ito M Komai K Mise-Omata S Iizuka-Koga M Noguchi Y Kondo T. Brain regulatory T cells suppress astrogliosis and potentiate neurological recovery. Nature. (2019) 565:246–50. 10.1038/s41586-018-0824-5 30602786

[51] Wasilewski D Priego N Fustero-Torre C Valiente M. Reactive astrocytes in brain metastasis. Front Oncol. (2017) 7:298. 10.3389/fonc.2017.00298 29312881 PMC5732246

[52] Placone AL Quinones-Hinojosa A Searson PC. The role of astrocytes in the progression of brain cancer: complicating the picture of the tumor microenvironment. Tumour Biol. (2016) 37:61–9. 10.1007/s13277-015-4242-0 26493995

[53] Valiente M Obenauf AC Jin X Chen Q Zhang XH Lee DJ. Serpins promote cancer cell survival and vascular co-option in brain metastasis. Cell. (2014) 156:1002–16. 10.1016/j.cell.2014.01.040 24581498 PMC3988473

[54] Klein A Schwartz H Sagi-Assif O Meshel T Izraely S Ben Menachem S. Astrocytes facilitate melanoma brain metastasis via secretion of IL-23. J Pathol. (2015) 236:116–27. 10.1002/path.4509 25639230

[55] Wang L Cossette SM Rarick KR Gershan J Dwinell MB Harder DR. Astrocytes directly influence tumor cell invasion and metastasis *in vivo*. PLoS ONE. (2013) 8:e80933. 10.1371/journal.pone.0080933 24324647 PMC3851470

[56] Stoletov K Strnadel J Zardouzian E Momiyama M Park FD Kelber JA. Role of connexins in metastatic breast cancer and melanoma brain colonization. J Cell Sci. (2013) 126:904–13. 10.1242/jcs.112748 23321642 PMC3625812

[57] Lin Q Balasubramanian K Fan D Kim SJ Guo L Wang H. Reactive astrocytes protect melanoma cells from chemotherapy by sequestering intracellular calcium through gap junction communication channels. Neoplasia. (2010) 12:748–54. 10.1593/neo.10602 20824051 PMC2933695

[58] Kim SJ Kim JS Park ES Lee JS Lin Q Langley RR. Astrocytes upregulate survival genes in tumor cells and induce protection from chemotherapy. Neoplasia. (2011) 13:286–98. 10.1593/neo.11112 21390191 PMC3050871

[59] Chen Q Boire A Jin X Valiente M Er EE Lopez-Soto A. Carcinoma-astrocyte gap junctions promote brain metastasis by cGAMP transfer. Nature. (2016) 533:493–8. 10.1038/nature18268 27225120 PMC5021195

[60] Schwartz H Blacher E Amer M Livneh N Abramovitz L Klein A. Incipient melanoma brain metastases instigate astrogliosis and neuroinflammation. Cancer Res. (2016) 76:4359–71. 10.1158/0008-5472.CAN-16-0485 27261506

[61] Priego N Zhu L Monteiro C Mulders M Wasilewski D Bindeman W STAT3 labels a subpopulation of reactive astrocytes required for brain metastasis. Nat Med. (2018) 24:1024–35. 10.1038/s41591-018-0044-4 29892069

[62] Ghoochani A Schwarz MA Yakubov E Engelhorn T Doerfler A Buchfelder M. MIF-CD74 signaling impedes microglial M1 polarization and facilitates brain tumorigenesis. Oncogene. (2016) 35:6246–61. 10.1038/onc.2016.160 27157615

[63] Heiland HD Ravi VM Behringer SP Frenking JH Wurm J Joseph K Tumor-associated reactive astrocytes aid the evolution of immunosuppressive environment in glioblastoma. Nat Commun. (2019) 10:2541 10.1038/s41467-019-10493-6 31186414 PMC6559986

[64] Bowman RL Klemm F Akkari L Pyonteck SM Sevenich L Quail DF. Macrophage ontogeny underlies differences in tumor-specific education in brain malignancies. Cell Rep. (2016) 17:2445–59. 10.1016/j.celrep.2016.10.052 27840052 PMC5450644

[65] Weinhard L di Bartolomei G Bolasco G Machado P Schieber NL Neniskyte U. Microglia remodel synapses by presynaptic trogocytosis and spine head filopodia induction. Nat Commun. (2018) 9:1228. 10.1038/s41467-018-03566-5 29581545 PMC5964317

[66] Ginhoux F Greter M Leboeuf M Nandi S See P Gokhan S. Fate mapping analysis reveals that adult microglia derive from primitive macrophages. Science. (2010) 330:841–5. 10.1126/science.1194637 20966214 PMC3719181

[67] Gomez Perdiguero E Klapproth K Schulz C Busch K Azzoni E Crozet L. Tissue-resident macrophages originate from yolk-sac-derived erythro-myeloid progenitors. Nature. (2015) 518:547–51. 10.1038/nature13989 25470051 PMC5997177

[68] Kierdorf K Erny D Goldmann T Sander V Schulz C Perdiguero EG. Microglia emerge from erythromyeloid precursors via Pu.1- and Irf8-dependent pathways. Nat Neurosci. (2013) 16:273–80. 10.1038/nn.3318 23334579

[69] Van Hove H Martens L Scheyltjens I De Vlaminck K Pombo Antunes AR De Prijck S. A single-cell atlas of mouse brain macrophages reveals unique transcriptional identities shaped by ontogeny and tissue environment. Nat Neurosci. (2019) 22:1021–35. 10.1038/s41593-019-0393-4 31061494

[70] Korin B Ben-Shaanan TL Schiller M Dubovik T Azulay-Debby H Boshnak NT. High-dimensional, single-cell characterization of the brain's immune compartment. Nat Neurosci. (2017) 20:1300–9. 10.1038/nn.4610 28758994

[71] Mrdjen D Pavlovic A Hartmann FJ Schreiner B Utz SG Leung BP High-dimensional single-cell mapping of central nervous system immune cells reveals distinct myeloid subsets in health, aging, and disease. Immunity. (2018) 48:599 10.1016/j.immuni.2018.02.014 29562204

[72] Goldmann T Wieghofer P Jordao MJ Prutek F Hagemeyer N Frenzel K. Origin, fate and dynamics of macrophages at central nervous system interfaces. Nat Immunol. (2016) 17:797–805. 10.1038/ni.3423 27135602 PMC4968048

[73] Chinnery HR Ruitenberg MJ McMenamin PG. Novel characterization of monocyte-derived cell populations in the meninges and choroid plexus and their rates of replenishment in bone marrow chimeric mice. J Neuropathol Exp Neurol. (2010) 69:896–909. 10.1097/NEN.0b013e3181edbc1a 20720507

[74] Mrdjen D Pavlovic A Hartmann FJ Schreiner B Utz SG Leung BP High-dimensional single-cell mapping of central nervous system immune cells reveals distinct myeloid subsets in health, aging, and disease. Immunity. (2018) 48:380–95.e6. 10.1016/j.immuni.2018.01.011 29426702

[75] Guneykaya D Ivanov A Hernandez DP Haage V Wojtas B Meyer N. Transcriptional and translational differences of microglia from male and female brains. Cell Rep. (2018) 24:2773–83.e6. 10.1016/j.celrep.2018.08.001 30184509

[76] Olah M Patrick E Villani AC Xu J White CC Ryan KJ. A transcriptomic atlas of aged human microglia. Nat Commun. (2018) 9:539. 10.1038/s41467-018-02926-5 29416036 PMC5803269

[77] Thion MS Low D Silvin A Chen J Grisel P Schulte-Schrepping J. Microbiome influences prenatal and adult microglia in a sex-specific manner. Cell. (2018) 172:500–16.e16. 10.1016/j.cell.2017.11.042 29275859 PMC5786503

[78] Villa A Gelosa P Castiglioni L Cimino M Rizzi N Pepe G. Sex-specific features of microglia from adult mice. Cell Rep. (2018) 23:3501–11. 10.1016/j.celrep.2018.05.048 29924994 PMC6024879

[79] Deczkowska A Keren-Shaul H Weiner A Colonna M Schwartz M Amit I. Disease-associated microglia: a universal immune sensor of neurodegeneration. Cell. (2018) 173:1073–81. 10.1016/j.cell.2018.05.003 29775591

[80] Keren-Shaul H Spinrad A Weiner A Matcovitch-Natan O Dvir-Szternfeld R Ulland TK. A unique microglia type associated with restricting development of Alzheimer's disease. Cell. (2017) 169:1276–90.e17. 10.1016/j.cell.2017.05.018 28602351

[81] Tay TL Sagar Dautzenberg J Grun D Prinz M. Unique microglia recovery population revealed by single-cell RNAseq following neurodegeneration. Acta Neuropathol Commun. (2018) 6:87. 10.1186/s40478-018-0584-3 30185219 PMC6123921

[82] Mathys H Adaikkan C Gao F Young JZ Manet E Hemberg M. Temporal tracking of microglia activation in neurodegeneration at single-cell resolution. Cell Rep. (2017) 21:366–80. 10.1016/j.celrep.2017.09.039 29020624 PMC5642107

[83] Masuda T Sankowski R Staszewski O Bottcher C Amann L Sagar. Spatial and temporal heterogeneity of mouse and human microglia at single-cell resolution. Nature. (2019) 566:388–92. 10.1038/s41586-019-0924-x 30760929

[84] Qiao S Qian Y Xu G Luo Q Zhang Z. Long-term characterization of activated microglia/macrophages facilitating the development of experimental brain metastasis through intravital microscopic imaging. J Neuroinflammation. (2019) 16:4. 10.1186/s12974-018-1389-9 30616691 PMC6323850

[85] Lorger M Felding-Habermann B. Capturing changes in the brain microenvironment during initial steps of breast cancer brain metastasis. Am J Pathol. (2010) 176:2958–71. 10.2353/ajpath.2010.090838 20382702 PMC2877856

[86] Chuang HN van Rossum D Sieger D Siam L Klemm F Bleckmann A. Carcinoma cells misuse the host tissue damage response to invade the brain. Glia. (2013) 61:1331–46. 10.1002/glia.22518 23832647 PMC3842117

[87] Wang L Wang Z Liu X Liu F. High-level C-X-C chemokine receptor type 4 expression correlates with brain-specific metastasis following complete resection of non-small cell lung cancer. Oncol Lett. (2014) 7:1871–6. 10.3892/ol.2014.1979 24932250 PMC4049707

[88] Salmaggi A Maderna E Calatozzolo C Gaviani P Canazza A Milanesi I. CXCL12, CXCR4 and CXCR7 expression in brain metastases. Cancer Biol Ther. (2009) 8:1608–14. 10.4161/cbt.8.17.9202 19625779

[89] Lee BC Lee TH Avraham S Avraham HK. Involvement of the chemokine receptor CXCR4 and its ligand stromal cell-derived factor 1alpha in breast cancer cell migration through human brain microvascular endothelial cells. Mol Cancer Res. (2004) 2:327–38. 15235108

[90] Phillips RJ Burdick MD Lutz M Belperio JA Keane MP Strieter RM. The stromal derived factor-1/CXCL12-CXC chemokine receptor 4 biological axis in non-small cell lung cancer metastases. Am J Respir Crit Care Med. (2003) 167:1676–86. 10.1164/rccm.200301-071OC 12626353

[91] Pyonteck SM Akkari L Schuhmacher AJ Bowman RL Sevenich L Quail DF. CSF-1R inhibition alters macrophage polarization and blocks glioma progression. Nat Med. (2013) 19:1264–72. 10.1038/nm.3337 24056773 PMC3840724

[92] Chen Z Feng X Herting CJ Garcia VA Nie K Pong WW. Cellular and molecular identity of tumor-associated macrophages in glioblastoma. Cancer Res. (2017) 77:2266–78. 10.1158/0008-5472.CAN-16-2310 28235764 PMC5741820

[93] Shinonaga M Chang CC Suzuki N Sato M Kuwabara T. Immunohistological evaluation of macrophage infiltrates in brain tumors. correlation with peritumoral edema. J Neurosurg. (1988) 68:259–65. 10.3171/jns.1988.68.2.0259 3276837

[94] Amit M Laider-Trejo L Shalom V Shabtay-Orbach A Krelin Y Gil Z. Characterization of the melanoma brain metastatic niche in mice and humans. Cancer Med. (2013) 2:155–63. 10.1002/cam4.45 23634283 PMC3639654

[95] Strik HM Stoll M Meyermann R. Immune cell infiltration of intrinsic and metastatic intracranial tumours. Anticancer Res. (2004) 24:37–42. 15015573

[96] Berghoff AS Lassmann H Preusser M Hoftberger R. Characterization of the inflammatory response to solid cancer metastases in the human brain. Clin Exp Metastasis. (2013) 30:69–81. 10.1007/s10585-012-9510-4 22752508

[97] Izraely S Ben-Menachem S Sagi-Assif O Telerman A Zubrilov I Ashkenazi O. The metastatic microenvironment: melanoma-microglia cross-talk promotes the malignant phenotype of melanoma cells. Int J Cancer. (2019) 144:802–17. 10.1002/ijc.31745 29992556

[98] Andreou KE Soto MS Allen D Economopoulos V de Bernardi A Larkin JR. Anti-inflammatory microglia/macrophages as a potential therapeutic target in brain metastasis. Front Oncol. (2017) 7:251. 10.3389/fonc.2017.00251 29164051 PMC5670100

[99] Xing F Liu Y Wu SY Wu K Sharma S Mo YY. Loss of XIST in breast cancer activates MSN-c-Met and reprograms microglia via exosomal mirna to promote brain metastasis. Cancer Res. (2018) 78:4316–30. 10.1158/0008-5472.CAN-18-1102 30026327 PMC6072593

[100] Sevenich L. Brain-resident microglia and blood-borne macrophages orchestrate central nervous system inflammation in neurodegenerative disorders and brain cancer. Front Immunol. (2018) 9:697. 10.3389/fimmu.2018.00697 29681904 PMC5897444

[101] Blazquez R Wlochowitz D Wolff A Seitz S Wachter A Perera-Bel J. PI3K: a master regulator of brain metastasis-promoting macrophages/microglia. Glia. (2018) 66:2438–55. 10.1002/glia.23485 30357946

[102] Tiwary S Morales JE Kwiatkowski SC Lang FF Rao G McCarty JH. Metastatic brain tumors disrupt the blood-brain barrier and alter lipid metabolism by inhibiting expression of the endothelial cell fatty acid transporter Mfsd2a. Sci Rep. (2018) 8:8267. 10.1038/s41598-018-26636-6 29844613 PMC5974340

[103] Yonemori K Tsuta K Ono M Shimizu C Hirakawa A Hasegawa T Disruption of the blood brain barrier by brain metastases of triple-negative and basal-type breast cancer but not HER2/neu-positive breast cancer. Cancer. (2010) 116:302–8. 10.1002/cncr.24735 19937674

[104] Avraham HK Jiang S Fu Y Nakshatri H Ovadia H Avraham S. Angiopoietin-2 mediates blood-brain barrier impairment and colonization of triple-negative breast cancer cells in brain. J Pathol. (2014) 232:369–81. 10.1002/path.4304 24421076

[105] Wu SY Watabe K. The roles of microglia/macrophages in tumor progression of brain cancer and metastatic disease. Front Biosci. (2017) 22:1805–29. 10.2741/4573 28410147 PMC5658785

[106] Lyle LT Lockman PR Adkins CE Mohammad AS Sechrest E Hua E. Alterations in pericyte subpopulations are associated with elevated blood-tumor barrier permeability in experimental brain metastasis of breast cancer. Clin Cancer Res. (2016) 22:5287–99. 10.1158/1078-0432.CCR-15-1836 27245829 PMC5093086

[107] Lockman PR Mittapalli RK Taskar KS Rudraraju V Gril B Bohn KA. Heterogeneous blood-tumor barrier permeability determines drug efficacy in experimental brain metastases of breast cancer. Clin Cancer Res. (2010) 16:5664–78. 10.1158/1078-0432.CCR-10-1564 20829328 PMC2999649

[108] Muller A Brandenburg S Turkowski K Muller S Vajkoczy P Resident microglia, and not peripheral macrophages, are the main source of brain tumor mononuclear cells. Int J Cancer. (2015) 137:278–88. 10.1002/ijc.29379 25477239

[109] Rietkotter E Bleckmann A Bayerlova M Menck K Chuang HN Wenske B. Anti-CSF-1 treatment is effective to prevent carcinoma invasion induced by monocyte-derived cells but scarcely by microglia. Oncotarget. (2015) 6:15482–93. 10.18632/oncotarget.3855 26098772 PMC4558165

[110] Jordao MJC Sankowski R Brendecke SM Sagar Locatelli G Tai YH. Single-cell profiling identifies myeloid cell subsets with distinct fates during neuroinflammation. Science. (2019) 363. 10.1126/science.aat7554 30679343

[111] Shemer A Grozovski J Tay TL Tao J Volaski A Suss P. Engrafted parenchymal brain macrophages differ from microglia in transcriptome, chromatin landscape and response to challenge. Nat Commun. (2018) 9:5206. 10.1038/s41467-018-07548-5 30523248 PMC6284018

[112] Haage V Semtner M Vidal RO Hernandez DP Pong WW Chen Z. Comprehensive gene expression meta-analysis identifies signature genes that distinguish microglia from peripheral monocytes/macrophages in health and glioma. Acta Neuropathol Commun. (2019) 7:20. 10.1186/s40478-019-0665-y 30764877 PMC6376799

[113] Bennett ML Bennett FC Liddelow SA Ajami B Zamanian JL Fernhoff NB. New tools for studying microglia in the mouse and human CNS. Proc Natl Acad Sci USA. (2016) 113:E1738–46. 10.1073/pnas.1525528113 26884166 PMC4812770

[114] Buttgereit A Lelios I Yu X Vrohlings M Krakoski NR Gautier EL. Sall1 is a transcriptional regulator defining microglia identity and function. Nat Immunol. (2016) 17:1397–406. 10.1038/ni.3585 27776109

[115] Mildner A Huang H Radke J Stenzel W Priller J. P2Y12 receptor is expressed on human microglia under physiological conditions throughout development and is sensitive to neuroinflammatory diseases. Glia. (2017) 65:375–87. 10.1002/glia.23097 27862351

[116] Konishi H Kobayashi M Kunisawa T Imai K Sayo A Malissen B. Siglec-H is a microglia-specific marker that discriminates microglia from CNS-associated macrophages and CNS-infiltrating monocytes. Glia. (2017) 65:1927–43. 10.1002/glia.23204 28836308

[117] Head JR Griffin WS. Functional capacity of solid tissue transplants in the brain: evidence for immunological privilege. Proc R Soc Lond B Biol Sci. (1985) 224:375–87. 10.1098/rspb.1985.0039 2862633

[118] Korn T Kallies A. T cell responses in the central nervous system. Nat Rev Immunol. (2017) 17:179–94. 10.1038/nri.2016.144 28138136

[119] D'Agostino PM Gottfried-Blackmore A Anandasabapathy N Bulloch K. Brain dendritic cells: biology and pathology. Acta Neuropathol. (2012) 124:599–614. 10.1007/s00401-012-1018-0 22825593 PMC3700359

[120] Russo MV McGavern DB. Immune surveillance of the CNS following infection and injury. Trends Immunol. (2015) 36:637–50. 10.1016/j.it.2015.08.002 26431941 PMC4592776

[121] Anandasabapathy N Victora GD Meredith M Feder R Dong B Kluger C. Flt3L controls the development of radiosensitive dendritic cells in the meninges and choroid plexus of the steady-state mouse brain. J Exp Med. (2011) 208:1695–705. 10.1084/jem.20102657 21788405 PMC3149213

[122] Harris MG Hulseberg P Ling C Karman J Clarkson BD Harding JS Immune privilege of the CNS is not the consequence of limited antigen sampling. Sci Rep. (2014) 4:4422 10.1038/srep04422 24651727 PMC3961746

[123] Aspelund A Antila S Proulx ST Karlsen TV Karaman S Detmar M. A dural lymphatic vascular system that drains brain interstitial fluid and macromolecules. J Exp Med. (2015) 212:991–9. 10.1084/jem.20142290 26077718 PMC4493418

[124] Louveau A Smirnov I Keyes TJ Eccles JD Rouhani SJ Peske JD. Structural and functional features of central nervous system lymphatic vessels. Nature. (2015) 523:337–41. 10.1038/nature14432 26030524 PMC4506234

[125] Raper D Louveau A Kipnis J. How do meningeal lymphatic vessels drain the CNS? Trends Neurosci. (2016) 39:581–6. 10.1016/j.tins.2016.07.001 27460561 PMC5002390

[126] Harter PN Bernatz S Scholz A Zeiner PS Zinke J Kiyose M. Distribution and prognostic relevance of tumor-infiltrating lymphocytes (TILs) and PD-1/PD-L1 immune checkpoints in human brain metastases. Oncotarget. (2015) 6:40836–49. 10.18632/oncotarget.5696 26517811 PMC4747372

[127] Berghoff AS Fuchs E Ricken G Mlecnik B Bindea G Spanberger T. Density of tumor-infiltrating lymphocytes correlates with extent of brain edema and overall survival time in patients with brain metastases. Oncoimmunology. (2016) 5:e1057388. 10.1080/2162402X.2015.1057388 26942067 PMC4760339

[128] Zakaria R Platt-Higgins A Rathi N Radon M Das S Das K. T-cell densities in brain metastases are associated with patient survival times and diffusion tensor MRI changes. Cancer Res. (2018) 78:610–6. 10.1158/0008-5472.CAN-17-1720 29212855 PMC5796648

[129] Mustafa DAM Pedrosa R Smid M van der Weiden M de Weerd V Nigg AL. T lymphocytes facilitate brain metastasis of breast cancer by inducing Guanylate-Binding Protein 1 expression. Acta Neuropathol. (2018) 135:581–99. 10.1007/s00401-018-1806-2 29350274 PMC5978929

[130] Alon R Kassner PD Carr MW Finger EB Hemler ME Springer TA. The integrin VLA-4 supports tethering and rolling in flow on VCAM-1. J Cell Biol. (1995) 128:1243–53. 10.1083/jcb.128.6.1243 7534768 PMC2120426

[131] Carman CV Martinelli R. T Lymphocyte-endothelial interactions: emerging understanding of trafficking and antigen-specific immunity. Front Immunol. (2015) 6:603. 10.3389/fimmu.2015.00603 26635815 PMC4657048

[132] Liebner S Dijkhuizen RM Reiss Y Plate KH Agalliu D Constantin G. Functional morphology of the blood-brain barrier in health and disease. Acta Neuropathol. (2018) 135:311–36. 10.1007/s00401-018-1815-1 29411111 PMC6781630

[133] Serres S Soto MS Hamilton A McAteer MA Carbonell WS Robson MD. Molecular MRI enables early and sensitive detection of brain metastases. Proc Natl Acad Sci USA. (2012) 109:6674–9. 10.1073/pnas.1117412109 22451897 PMC3340084

[134] Soto MS Serres S Anthony DC Sibson NR. Functional role of endothelial adhesion molecules in the early stages of brain metastasis. Neuro Oncol. (2014) 16:540–51. 10.1093/neuonc/not222 24311639 PMC3956349

[135] Taggart D Andreou T Scott KJ Williams J Rippaus N Brownlie RJ. Anti-PD-1/anti-CTLA-4 efficacy in melanoma brain metastases depends on extracranial disease and augmentation of CD8(+) T cell trafficking. Proc Natl Acad Sci USA. (2018) 115:E1540–9. 10.1073/pnas.1714089115 29386395 PMC5816160

[136] Woods AN Wilson AL Srivinisan N Zeng J Dutta AB Peske JD. Differential expression of homing receptor ligands on tumor-associated vasculature that control CD8 effector T-cell entry. Cancer Immunol Res. (2017) 5:1062–73. 10.1158/2326-6066.CIR-17-0190 29097419 PMC6069521

[137] Spranger S Luke JJ Bao R Zha Y Hernandez KM Li Y. Density of immunogenic antigens does not explain the presence or absence of the T-cell-inflamed tumor microenvironment in melanoma. Proc Natl Acad Sci USA. (2016) 113:E7759–68. 10.1073/pnas.1609376113 27837020 PMC5137753

[138] Mansfield AS Ren H Sutor S Sarangi V Nair A Davila J. Contraction of T cell richness in lung cancer brain metastases. Sci Rep. (2018) 8:2171. 10.1038/s41598-018-20622-8 29391594 PMC5794798

[139] Ogiya R Niikura N Kumaki N Yasojima H Iwasa T Kanbayashi C. Comparison of immune microenvironments between primary tumors and brain metastases in patients with breast cancer. Oncotarget. (2017) 8:103671–81. 10.18632/oncotarget.22110 29262592 PMC5732758

[140] Dudley ME Wunderlich JR Yang JC Sherry RM Topalian SL Restifo NP. Adoptive cell transfer therapy following non-myeloablative but lymphodepleting chemotherapy for the treatment of patients with refractory metastatic melanoma. J Clin Oncol. (2005) 23:2346–57. 10.1200/JCO.2005.00.240 15800326 PMC1475951

[141] Farber SH Tsvankin V Narloch JL Kim GJ Salama AK Vlahovic G. Embracing rejection: immunologic trends in brain metastasis. Oncoimmunology. (2016) 5:e1172153. 10.1080/2162402X.2016.1172153 27622023 PMC5006920

[142] Sugihara AQ Rolle CE Lesniak MS. Regulatory T cells actively infiltrate metastatic brain tumors. Int J Oncol. (2009) 34:1533–40. 10.3892/ijo_00000282 19424570

[143] Jacobs JF Idema AJ Bol KF Nierkens S Grauer OM Wesseling P. Regulatory T cells and the PD-L1/PD-1 pathway mediate immune suppression in malignant human brain tumors. Neuro Oncol. (2009) 11:394–402. 10.1215/15228517-2008-104 19028999 PMC2743219

[144] Berghoff AS Venur VA Preusser M Ahluwalia MS. Immune checkpoint inhibitors in brain metastases: from biology to treatment. Am Soc Clin Oncol Educ Book. (2016) 35:e116–22. 10.14694/EDBK_100005 27249713

[145] Cohen JV Kluger HM. Systemic immunotherapy for the treatment of brain metastases. Front Oncol. (2016) 6:49. 10.3389/fonc.2016.00049 27014624 PMC4783384

[146] Harary M Reardon DA Iorgulescu JB. Efficacy and safety of immune checkpoint blockade for brain metastases. CNS Oncol. (2019). 10.2217/cns-2018-0018. [Epub ahead of print]. 30854898 PMC6713022

[147] Milsch L Gesierich A Kreft S Livingstone E Zimmer L Goebeler M. Patterns of disease control and survival in patients with melanoma brain metastases undergoing immune-checkpoint blockade. Eur J Cancer. (2018) 99:58–65. 10.1016/j.ejca.2018.05.012 29906735

[148] Ajithkumar T Parkinson C Fife K Corrie P Jefferies S. Evolving treatment options for melanoma brain metastases. Lancet Oncol. (2015) 16:e486–97. 10.1016/S1470-2045(15)00141-2 26433822

[149] Margolin K. Ipilimumab in a Phase II trial of melanoma patients with brain metastases. Oncoimmunology. (2012) 1:1197–9. 10.4161/onci.20687 23170278 PMC3494644

[150] Long GV Atkinson V Lo S Sandhu S Guminski AD Brown MP. Combination nivolumab and ipilimumab or nivolumab alone in melanoma brain metastases: a multicentre randomised phase 2 study. Lancet Oncol. (2018) 19:672–81. 10.1016/S1470-2045(18)30139-6 29602646

[151] Tawbi HA Forsyth PA Algazi A Hamid O Hodi FS Moschos SJ. Combined nivolumab and ipilimumab in melanoma metastatic to the brain. N Engl J Med. (2018) 379:722–30. 10.1056/NEJMoa1805453 30134131 PMC8011001

[152] Park B Yee C Lee KM. The effect of radiation on the immune response to cancers. Int J Mol Sci. (2014) 15:927–43. 10.3390/ijms15010927 24434638 PMC3907847

[153] Sevenich L. Turning “cold” into “hot” tumors-opportunities and challenges for radio-immunotherapy against primary and metastatic brain cancers. Front Oncol. (2019) 9:163. 10.3389/fonc.2019.00163 30941312 PMC6433980

[154] Mouw KW Goldberg MS Konstantinopoulos PA D'Andrea AD. DNA damage and repair biomarkers of immunotherapy response. Cancer Discov. (2017) 7:675–93. 10.1158/2159-8290.CD-17-0226 28630051 PMC5659200

[155] Lehrer EJ McGee HM Peterson JL Vallow L Ruiz-Garcia H Zaorsky NG. Stereotactic radiosurgery and immune checkpoint inhibitors in the management of brain metastases. Int J Mol Sci. (2018) 19:3054. 10.3390/ijms19103054 30301252 PMC6213912

[156] Eagles ME Nassiri F Badhiwala JH Suppiah S Almenawer SA Zadeh G. Dendritic cell vaccines for high-grade gliomas. Ther Clin Risk Manag. (2018) 14:1299–313. 10.2147/TCRM.S135865 30100728 PMC6067774

[157] Martinez M Moon EK. CAR T cells for solid tumors: new strategies for finding, infiltrating, and surviving in the tumor microenvironment. Front Immunol. (2019) 10:128. 10.3389/fimmu.2019.00128 30804938 PMC6370640

[158] Priceman SJ Tilakawardane D Jeang B Aguilar B Murad JP Park AK. Regional delivery of chimeric antigen receptor-engineered T cells effectively targets HER2(+) breast cancer metastasis to the brain. Clin Cancer Res. (2018) 24:95–105. 10.1158/1078-0432.CCR-17-2041 29061641 PMC7685198

[159] Ries CH Cannarile MA Hoves S Benz J Wartha K Runza V. Targeting tumor-associated macrophages with anti-CSF-1R antibody reveals a strategy for cancer therapy. Cancer Cell. (2014) 25:846–59. 10.1016/j.ccr.2014.05.016 24898549

[160] Dagher NN Najafi AR Kayala KM Elmore MR White TE Medeiros R. Colony-stimulating factor 1 receptor inhibition prevents microglial plaque association and improves cognition in 3xTg-AD mice. J Neuroinflammation. (2015) 12:139. 10.1186/s12974-015-0366-9 26232154 PMC4522109

[161] Stafford JH Hirai T Deng L Chernikova SB Urata K West BL. Colony stimulating factor 1 receptor inhibition delays recurrence of glioblastoma after radiation by altering myeloid cell recruitment and polarization. Neuro Oncol. (2016) 18:797–806. 10.1093/neuonc/nov272 26538619 PMC4864255

[162] Dere E Zlomuzica A. The role of gap junctions in the brain in health and disease. Neurosci Biobehav Rev. (2012) 36:206–17. 10.1016/j.neubiorev.2011.05.015 21664373

